# Metabolite Profiling of Microwave-Assisted *Sargassum fusiforme* Extracts with Improved Antioxidant Activity Using Hybrid Response Surface Methodology and Artificial Neural Networking-Genetic Algorithm

**DOI:** 10.3390/antiox11112246

**Published:** 2022-11-14

**Authors:** Ahsan Javed, Marufa Naznin, Md. Badrul Alam, Alshammari Fanar, Bo-Rim Song, Sunghwan Kim, Sang-Han Lee

**Affiliations:** 1 Department of Food Science and Biotechnology, Graduate School, Kyungpook National University, Daegu 41566, Republic of Korea; 2 Department of Chemistry, Kyungpook National University, Daegu 41566, Republic of Korea; 3 Food and Bio-Industry Research Institute, Inner Beauty/Antiaging Center, Kyungpook National University, Daegu 41566, Republic of Korea; 4 Mass Spectroscopy Converging Research Center, Green Nano Materials Research Center, Kyungpook National University, Daegu 41566, Republic of Korea

**Keywords:** *Sargassum fusiforme*, microwave-assisted extraction, secondary metabolites, antioxidant activity

## Abstract

*Sargassum fusiforme* (SF) is a popular edible brown macroalga found in Korea, Japan, and China and is known for its health-promoting properties. In this study, we used two sophisticated models to obtain optimized conditions for high antioxidant activity and metabolite profiling using high-resolution mass spectrometry. A four-factor central composite design was used to optimize the microwave-assisted extraction and achieve the maximum antioxidant activities of DPPH (Y_1_: 28.01 % inhibition), ABTS (Y_2_: 36.07 % inhibition), TPC (Y_3_: 43.65 mg GAE/g), and TFC (Y_4_: 17.67 mg CAE/g), which were achieved under the optimized extraction conditions of X_1_: 47.67 %, X_2_: 2.96 min, X_3_: 139.54 °C, and X_4_: 600.00 W. Moreover, over 79 secondary metabolites were tentatively identified, of which 12 compounds were reported for the first time in SF, including five phenolic (isopropyl 3-(3,4-dihydroxyphenyl)-2-hydroxypropanoate, 3,4-dihydroxyphenylglycol, scopoletin, caffeic acid 4-sulfate, and cinnamoyl glucose), two flavonoids (4’,7-dihydroxyisoflavone and naringenin), three phlorotannins (diphlorethohydroxycarmalol, dibenzodioxin-1,3,6,8-tetraol, and fucophlorethol), and two other compounds (dihydroxyphenylalanine and 5-hydroxybenzofuran-2(3H)-one) being identified for the first time in optimized SF extract. These compounds may also be involved in improving the antioxidant potential of the extract. Therefore, optimized models can provide better estimates and predictive capabilities that would assist in finding new bioactive compounds with improved biological activities that can be further applied at a commercial level.

## 1. Introduction

Phenolic compounds, which exhibit numerous biological and pharmacological activities, are among the most interesting in plant fractions [1]. Phenolic compounds from red, green, and brown seaweed have numerous health-promoting properties, including antioxidant, anticancer, antiviral, and anti-inflammatory activities [2]. Among brown seaweeds, *Sargassum fusiforme* (SF) belongs to the family Sargassaceae and is widely found in the coastal areas of Asia, especially in Korea, China, and Japan [3]. In Korea and Japan, like other marine organisms, SF is consumed as a sea vegetable because it possesses numerous nutritional components, such as proteins, minerals, phenolic compounds, dietary fibers, and polysaccharides [4,5]. The potential applications of these bioactive moieties in the pharmaceutical, food, and chemical industries have led to growing interest in the development and optimization of extraction techniques to isolate bioactive moieties from natural sources, as extraction is the most important step that plays a significant role in the quantity and quality of the result [6,7,8].

Recent advancements in extraction techniques have led to the development of efficient and new approaches for extracting natural bioactive components with improved yields and lower extraction times, temperatures, and solvent use. Numerous non-conventional techniques, such as ultrasound-assisted extraction (UAE) [9,10], pressurized liquid extraction (PLE) [11], supercritical fluid extraction (SFE) [12], and microwave-assisted extraction (MAE) [13], fulfill these requirements. In comparison with other techniques, MAE has its advantages, such as low cost and lower extraction time; thus, it can be categorized as “green technology” according to environmental policies [14,15]. The extraction time, temperature, technique, solid-to-liquid ratio, and solvent concentration are significant variables that, individually or in combination, alter the extraction efficiency. Optimizing the extraction conditions using a traditional methodology (one factor at a time) is a difficult, laborious, and time-consuming procedure. Empirical methods based on statistical or artificial intelligence methodologies have therefore been applied to overcome this problem [10]. RSM is a sophisticated statistical and mathematical approach used in the engineering field to optimize and improve conditions by analyzing the interactions between independent variables and response variables [16]. In RSM, a non-linear equation is used along with other designs, that is, the central composite design (CCD) or Box–Behnken design, to fit the experimental responses. Artificial neural networking (ANN) is a computational and mathematical approach that works based on the principles of the human brain. ANN, in comparison with RSM, is considered a superior and powerful tool because of its ability to adapt itself from the observations and predict the outcome after training on the experimental responses [17]. Moreover, optimized extraction conditions in combination with analytical tools such as high-resolution liquid chromatography-mass spectrometry (HR-LC-MS) have enabled the tentative identification of bioactive compounds in different species.

Previously, there was limited literature available that only focused on the optimization of polysaccharides of SF using RSM [18] without using ANN and green extraction technology, whereas in another trial, Li et al. [19] extracted and identified the phlorotannin fraction without using non-conventional extraction techniques and also without sophisticated statistical models. Therefore, in order to overcome the gaps, the present study was designed to optimize MAE-SF extracts using two sophisticated statistical tools (RSM and ANN) to obtain optimal conditions for exhibiting higher antioxidant activity and to determine the presence of bioactive compounds in SF using high-resolution tandem mass spectrometry (HR-LC-MS).

## 2. Materials and Methods

### 2.1. Chemicals and Reagents

Chemicals, reagents, and standards were procured from Sigma-Aldrich (St. Louis, MO, USA). Anhydrous sodium carbonate and acetic acid were acquired from Ducksan Pure Chemicals (Ansan, Republic of Korea), while sodium hydroxide and aluminum chloride hexahydrate were procured from Junsei Chemical (Tokyo, Japan) and Samchun Pure Chemical (Pyeongtaek, Republic of Korea), respectively. Acetic acid and anhydrous sodium carbonate were purchased from Ducksan Pure Chemicals (Ansan, Republic of Korea).

### 2.2. Sample Collection and Preparation

SF was collected from the coastal areas of Korea in mid-May 2021 and confirmed by a specialist at the Department of Oceanography, Kyungpook National University, Korea. Samples were washed, dried for four days at 37 °C, ground to obtain powder (approximately 800 g), and stored at −20 °C in the polyethylene bags for further analysis.

### 2.3. MAE Procedure

MAE was performed following the protocol of Kashif et al. [20] using a microwave-assisted extractor (MARS 6; CEM, Matthews, NC, USA) fitted with 50 mL quartz vessels with some minor modifications. Briefly, the SF sample was extracted three times by adjusting the microwave power (150–750 W) and time. Ten g of SF powder was placed in 500 mL Erlenmeyer flasks, and aqueous ethanol (150 mL) was added at different concentrations (25–75%) 30 min before extraction. Extraction was performed using CCD (Supplementary Table S1). After extraction, flasks were allowed to cool at room temperature for 1 h and were filtered (Whatman No. 1; Schleicher & Schuell, Keene, NH, USA), followed by concentration to dryness in a rotary evaporator (Tokyo Rikakikai, Tokyo, Japan) at 40 °C and 50 rpm. In addition, samples were lyophilized using a freezer dryer and stored at −20 °C in Falcon tubes (15 mL) for further analysis.

### 2.4. Antioxidant Activities

Folin-Ciocalteu reagent was used to assess the total polyphenolic content (TPC) using gallic acid as a reference curve. It is a rapid and easy procedure based on the association between the detected color change and reactive reagent attenuation as a result of phenolic compounds present in the plant matrix. Total flavonoid content (TFC) was determined using the aluminum chloride colorimetric procedure [21]. Similarly, the radical scavenging activity of the SF extracts was assessed using 2,2-diphenyl-1-picrylhydrazyl (DPPH) and 2,2-azino-bis-3-ethylbenzothiazoline-6-sulfonic acid (ABTS) [22].

### 2.5. Statistical Modeling and Data Calculation

#### 2.5.1. Response Surface Methodology (RSM)

The RSM was implemented to assess the best possible combination of independent parameters to maximize the output of the dependent variables. The dependent variables DPPH, ABTS, TPC, and TFC were calculated in triplicate, and mean values were employed for regression analysis, as shown in Table 1. Experimental results were analyzed using Design Expert software to predict the optimal extraction conditions, and a second-order polynomial model Equation (1) was used to fit the model between independent and dependent variables. Various other tests, including the coefficient of variation (CV), adjusted coefficient of determination (Adj. R^2^), lack of fit, and coefficient of determination (R^2^), were employed to validate the RSM model [23].

(1) $$y=\beta_{0}+\sum_{i=1}^{z}\beta_{i}X_{i}+\sum_{i=1}^{z}\beta_{ii}X_{i}^{2}+\sum_{j}\sum_{<i=2}^{z}\beta_{ij}X_{j}X_{i}+\varepsilon_{j}$$

where *y* represents the predicted variables; *β*_0_, *β_i_*, *β_ii_*, and *β_ij_* represent the regression coefficients of the intercept coefficient, linear, quadratic, and second-order terms, respectively; *X_i_* and *X_j_* are the independent variables (*j* and *I* range from 1*–z*), *z* is the number of factors (*z* = 4); *ε_j_* is the error.

The Design Expert software package (11 STAT-EASE, Minneapolis, MN, USA) was used for graphical, regression, and experimental designs. ANOVA was used to determine the adequacy of the fitted model, the significance of the independent variables, their association, and the statistical significance of the regression coefficients.

#### 2.5.2. ANN

**Figure 1:**
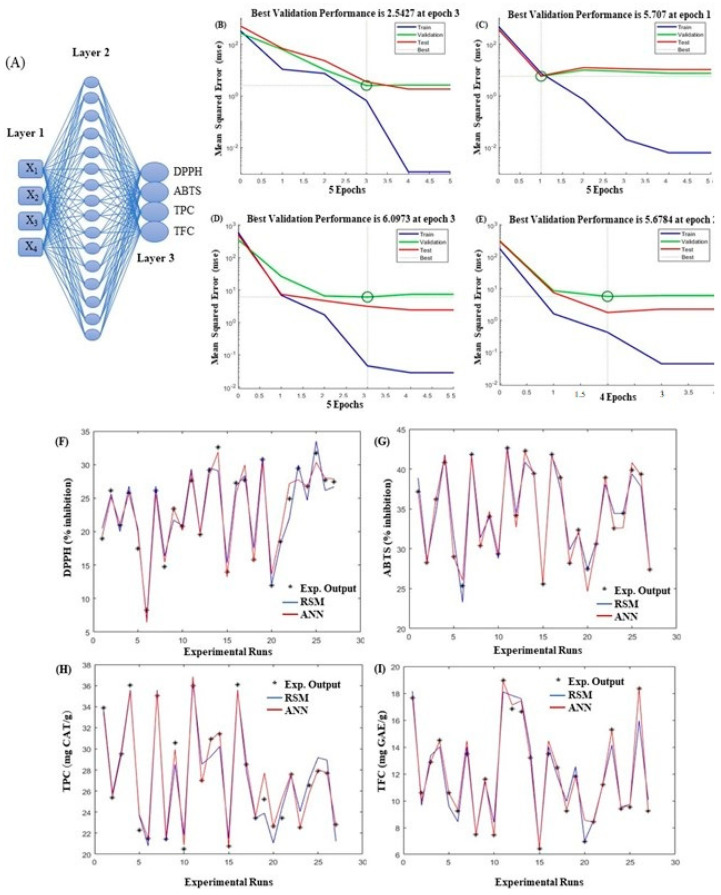
Optimal topology of optimized MAE-assisted SF extracts. Best architecture of a developed MAE-ANN model with the lowest mean square error (MSE) (**A**); The results of Levenberg-Marquardt algorithm with optimum numbers of neurons for best validation performance compared with training, testing and validation data for dependent variables DPPH (**B**) ABTS (**C**), TPC (**D**), and TFC (**E**), and comparison among experiment run (*), RSM (blue line), and ANN (red line) for DPPH (**F**), ABTS (**G**), TPC (**H**), and TFC (**I**) using deep learning toolbox MATLAB software.

ANN is a statistical machine learning technique that predicts nonlinear relationships between dependent variables and input parameters [24]. The model was constructed using the deep learning toolbox MATLAB R2020a (Mathworks, Minneapolis, MA, USA). The model was trained, tested, and validated using the experimental outputs obtained from the 27 experimental runs (Table 1). The multilayer perceptron topology is comprised of three layers. The first (input) layer contains four independent parameters, and the middle layer comprises 16 neurons to achieve the optimal conditions to obtain the maximum output results. The third (output) layer contains four dependent variables, as shown in Figure 1A. The hit and trial technique was adopted to train the data and to lower the mean square error (MSE) calculated from Equation (2). The feed-forward and cascade feed-forward networks with the Broyden—Fletcher—Goldfarb—Shanno (BFGS) and Levenberg—Marquardt back-propagation (trainlm) algorithms were used [21,25]. The model exhibits a higher coefficient of determination (R^2^) calculated from Equation (3), a lower standard error of prediction (SEP) from Equation (4), absolute average deviation (AAD) from Equation (5), and root mean squared error (RMSE) from Equation (6), which are considered optimal ANN models.

(2) $$\mathrm{MSE}=\frac{1}{N}\sum_{i=1}^{N}{\left(Y_{\mathrm{ANN}}-Y_{Exp}\right)}^{2}\;$$

where *Y_Exp_* is the experimental data, *Y_ANN_* is the predicted value, and *N* is the number of samples.

(3) $$R^{2}=1-\frac{\sum_{i=1}^{n}{\left(x_{i}-x_{ik}\right)}^{2}}{\sum_{i=1}^{n}{\left(x_{ik}-x_{z}\right)}^{2}}$$

(4) $$\mathrm{RMSE}=\sqrt{\frac{1}{n}\sum_{i=1}^{n}{\left(x_{i}-x_{ik}\right)}^{2}}$$

(5) $$\mathrm{ADD}\;\%=\left[\frac{\sum_{i=1}^{n}\left(\left|x_{ik}-x_{i}\right|/x_{ik}\right)}{n}\right]\times 100$$

(6) $$\mathrm{SEP}\;\%=\frac{\mathrm{RMSE}}{y_{m}}\times 100$$

### 2.6. Process Optimization Using RSM-GA, ANN-GA and RSM-DF

To optimize the MAE process, three different techniques were applied to the developed models, i.e., RSM-GA, RSM-DF (desirability functions), and ANN-GA. Data from the developed models (ANN and RSM) were subjected to GA (as initial input) using the optimization toolbox of MATLAB R2020a (Mathworks, Inc., Minneapolis, MA, USA). In GA, the RSM and ANN data were added as a fitness function to achieve the highest results for all the target responses. The initial population size, mutation fraction, evolutionary algebra, and crossover percentage were selected based on the current situation, while the remaining parameters were left at their default settings. The objective function was trained using the model data generated by the RSM-ANN, and the GA was implemented by maximizing the problems [26]. The RSM-DF was achieved by using the “Design Expert” software.

### 2.7. Cell Viability Assay

The effects of SF extracts on RAW 264.7 cells (American Type Culture Collection, Manassas, VA, USA) were determined using 3-(4,5-dimethylthiazol-2-yl)-2,5-diphenyltetrazolium bromide (MTT) assays described by [27]. Briefly, RAW 264.7 cells were seeded in a 96-well plate and incubated at 37 °C for 18 h. LPS (1 μg/mL) was induced in cells with SF extracts (10–30 mg) and incubated further for 20 h. The medium was sucked and, before incubation at 37 °C for 1 h, cells were treated with 100 μL MTT (10%) solution. Then, 100% dimethyl sulfoxide (DMSO) was added after the removal of the solution, and optical density (OD) was calculated at 590 nm using a microplate reader (Victor3, PerkinElmer, Waltham, MA, USA).

### 2.8. Intracellular Reactive Oxygen Species (ROS) Measurement

ROS generation as a result of cellular oxidative stress was assessed using the DCFH-DA method following the protocol of [28]. Briefly, RAW 264.7 cells were seeded for 24 h and then treated with samples at different concentrations. After 1 h, the cells were treated with 2, 2′-azobis (2-amidinopropane) dihydrochloride (AAPH) and incubated again for 30 min. With the help of PBS, cells were washed followed by treatment with DCFH-DA (25 µM), the plate was placed in the incubator at 37 °C for 30 min. The fluorescence intensity was determined at both the excited (485 nm) and emitted states (528 nm) using a Victor fluorescence microplate reader (PerkinElmer).

### 2.9. Western Blotting and Cell Lysate Preparation

Cell lysates were prepared following the standard methodology and then treated with 5X SDS-PAGE (3M Science, Seoul, Republic of Korea) sample buffer, and proteins were denatured by heating it at 95 °C for 10 min. Proteins were separated according to their molecular weight using 10% SDS gel electrophoresis, followed by the transfer of bands to nitrocellulose membranes for 2 h. A 5% bovine serum albumin (BSA) was added to the membrane and incubated overnight at 4 °C with the first antibody, followed by incubation with a secondary antibody (anti-goat IgG). Bands were detected using a chemiluminescence system (PerkinElmer) [29].

### 2.10. Compounds Identification Using ESI-MS/MS

The SF extract was analyzed to profile the secondary metabolites by following the protocol described by Choi et al. [21] using a Q-Executive Orbitrap mass spectrometer (Thermo Fisher Scientific, San Jose, CA, USA). Briefly, the SF sample was inserted at a rate of 15 μL/min using a 500 μL graduated syringe (Hamilton, Reno, NV, USA) and a syringe pump (Model 11, Harvard, Holliston, MA, USA) in the ESI source. The other parameters of ESI-MS (negative mode) were set as follows: the flow rates of auxiliary gas, sheath gas, and seep gas were set at 0, 5, and 0, respectively, spray voltage was set at 4.20 kV, capillary temperature was set at 320 °C, mass resolution of 140,000 (full width at half maximum, FWHM), S-lens at Rf level, and automatic gain control of 5 × 10^6^. Three different normalized collision energies (10, 30, and 40 eV) were applied stepwise using the same instrument [30].

### 2.11. Data Processing

The mass spectral data was analyzed with Xcalibur 3.1 with Foundation 3.1 (Thermo Fisher Scientific, Rockford, IL, USA). The detected m/z peaks were identified by comparing the exact masses of monoisotopic (negative mode) masses and ESI-MS/MS breakage patterns from an online database and as in-house MS/MS database. The online databases included FooDB [31], METLIN [32], and CFM-ID 4.0 [33].

### 2.12. Statistical Analysis

The final results were analyzed using MATLAB and Design Expert 11 software. All data is presented as the mean ± standard deviation of at least three independent experiments (n = 3), where each experiment had a minimum of three replicates for each sample. Differences were considered significant at *p* < 0.001, *p* < 0.01, and *p* < 0.05.

## 3. Results and Discussion

### 3.1. RSM Modeling

Table 1 shows a comparison between dependent response variables (DPPH, ABTS, TPC, and TFC) and predicted outcomes of RSM and ANN for all 27 runs obtained after MAE according to CCD as shown in Figure 1C. The DPPH, ABTS, TPC, and TFC contents of SF extracts varied in the ranges of 7.31–32.74% inhibition, 25.36–42.61% inhibition, 20.48–36.08 mg GAE/g, and 6.94–18.95 mg CAE/g, respectively. The ANOVA results are depicted in Table 2. For a well-fitted model, R^2^ values should be close to 1, and, in our trial, all four variables, that is, DPPH (0.95), ABTS (0.96), TPC (0.95), and TFC (0.97), exhibited higher R^2^ values, which shows that our model fits the second-order polynomial equation well. Similarly, other factors, including the CV, regression coefficient (β), F-value, adjusted correlation factor (R^2^), adequate precision, and lack of fit (probability), also contributed to the significance of the model. In the current study, lack of fit (0.09–0.35), F-values (2.18–9.58), Adj R^2^ (0.89–0.94), adequate precision (12.92–19.08), and CV (4.81–8.90) indicated that the fitness of the RSM model outcomes are in line with Simic et al. [6], as they checked the total polyphenolic compounds in the chokeberries using RSM and ANN. Likewise, Ameer et al. [26] also checked the parameters, including (R^2^, CV, adeq. Precision) during the recovery of stevioside and rebaudioside-A from *Stevia rebaudiana* leaves using MAE, which also supports our results. High F-values (*p* < 0.05) indicate that the lack of fit is non-significant, and our RSM model had higher F-values, showing that it was well-fitted, and these results are similar to Choi et al. [21].

#### 3.1.1. Effect of MAE-SF Parameters on DPPH and ABTS

To better understand the association between the independent and response variables, contour plots were produced, as shown in Figure S1. As shown in Table 2, linear terms of X_3_ exerted negative effects on ABTS and DPPH. In quadratic terms, X_2_^2^ and X_3_^2^ hurt DPPH, whereas, for ABTS, all four quadratic terms showed significant negative effects. In terms of interactions, X_1_X_2_, X_2_X_4_, and X_3_X_4_ had negative effects on both ABTS and DPPH. The maximum activity (32.74 mg GAE/g) for DPPH was calculated at run 25 (X_1_: 30%, X_2_: 4 min, X_3_: 110 °C, X_4_: 600 W), whereas the ABTS highest activity (42.61 mg CAE/g) was recorded at run 11 (X_1_: 50%, X_2_: 3 min, X_3_: 130 °C, X_4_: 750 W). The lack of fit for DPPH (0.1138) and ABTS (0.0981) and the coefficients of regression of 0.9543 and 0.9608 for DPPH and ABTS, respectively, suggest that the RSM model fits well. The fitted second-order polynomial equations for DPPH (% inhibition) and ABTS (% inhibition) are shown in Equations (7) and (8) as follows:

(7) $$\begin{array}{c} Y_{1}=26.34+0.6782X_{1}+1.72X_{2}-3.42X_{3}+1.245X_{4}-2.99X_{1}X_{2}+0.2740X_{1}X_{3}+2.45X_{1}X_{4}+0.1471X_{2}X_{3}\\ -1.02X_{2}X_{4}-1.43X_{3}X_{4}+0.6328X_{1}^{2}-1.07X_{2}^{2}-3.42X_{3}^{2}+0.2118X_{4}^{2} \end{array}$$

(8) $$\begin{array}{c} Y_{2}=41.84+0.9626X_{1}+0.4558X_{2}-2.40X_{3}+3.13X_{4}-0.6606X_{1}X_{2}+1.44X_{1}X_{3}+0.4581X_{1}X_{4}+1.08X_{2}X_{3}\\ +1.34X_{2}X_{4}-0.4356X_{3}X_{4}-1.47X_{1}^{2}-1.72X_{2}^{2}-3.60X_{3}^{2}-1.54X_{4}^{2} \end{array}$$

#### 3.1.2. Effect of MAE-SF Parameters on TPC and TFC

Similar to DPPH and ABTS, contour plots were generated for TPC and TFC to determine the relationship between the independent variables by varying the conditions. TPC (36.06 mg GAE/g) and TFC (16.85) exhibited the highest activities in runs 16 and 12, respectively (Table 1). ANOVA results show that TPC had significant effects in terms of linear (X_1_, X_3_, X_4_), interaction (X_1_X_3_, X_2_X_3_, X_2_X_4_), and quadratic relationships (X_1_^2^, X_2_^2^, X_3_^2^, X_4_^2^). The TFC exhibited significant effects in terms of linear (X_1_, X_2_, X_3_, X_4_), interaction (X_1_X_2_, X_1_X_3_, X_2_X_3_, X_2_X_4_, X_3_X_4_), and quadratic relationships (X_2_^2^, X_3_^2^) (Table 2). Likewise, the lack of fit for TPC (0.1025) and TFC (0.3556), along with the coefficient of determination for TPC (0.9533) and TFC (0.9745), show that our model could be fitted well. The relationship between TPC, TFC independent, and response variables can be determined using Equations (9) and (10), as follows:

(9) $$\begin{array}{ll} Y_{3}=35.48-0.980X_{1} & +0.1990X_{2}-1.19X_{3}+1.99X_{4}+0.1952X_{1}X_{2}\\ & +1.06X_{1}X_{3}+0.7562X_{1}X_{4}+1.41X_{2}X_{3}+1.94X_{2}X_{4}\\ & +0.4797X_{3}X_{4}-2.42X_{1}^{2}-2.58X_{2}^{2}-3.33X_{3}^{2}-1.13X_{4}^{2} \end{array}$$

(10) $$\begin{array}{ll} Y_{4}=13.26-0.4032X_{1} & +1.677X_{2}-0.6597X_{3}+1.51X_{4}-0.5511X_{1}X_{2}\\ & +1.01X_{1}X_{3}+0.1127X_{1}X_{4}+1.11X_{2}X_{3}+1.80X_{2}X_{4}\\ & +0.6715X_{3}X_{4}+0.1636X_{1}^{2}-0.4717X_{2}^{2}-1.15X_{3}^{2}+0.0652X_{4}^{2} \end{array}$$

### 3.2. ANN Modeling

The ANN architecture topology for MAE-SF conditions is shown in Figure 1. For a well-developed model, the testing and training errors should be minimal, and the epochs should be kept optimal. Lower epoch levels result in an under-fitted model, whereas high epochs result in model over-fitting. In this study, an ANN topology of 4–16–4 was found to be sufficient, with the least MSE and a higher R^2^ for better model reliability and precision, as shown in Figure S2. Figure 1B–E shows the performance of DPPH, ABTS, TPC, and TFC, which gradually reached the best validation performance of 2.54 at epoch 3, 5.70 at epoch 1, 6.09 at epoch 3, and 5.67 at epoch 2, respectively. Figure 1F–I shows a comparison between the experimental response and both the RSM and ANN predictions. Our outcomes are inconsistent with the results reported by Ameer et al. [26] for the recovery of bioactive components from *Stevia rebaudiana* leaf extracts using ultrasound-assisted extraction conditions with a hybrid RSM-ANN-GA model. Table 3 presents the model validation parameters. For an optimal model, the values of RMSE, SEP, and AAD should be lower, whereas R^2^ should be higher. In our study, for all four response variables, SEP, RMSE, and AAD values were lower, whereas R^2^ was higher in the ANN model than in the RSM model, which validated the superiority of the ANN model.

### 3.3. Process Optimization and Model Validation

The data generated by the RSM and ANN models was used to optimize the extraction conditions by using a genetic algorithm. A scalar technique was used in the objective function instead of using second-or first-order derivatives [34]. GA is adopted by maximizing the outputs of RSM and ANN in the objective function. For the fitness function, the rank method was used, whereas a stochastic uniform was utilized for the selection method. In GA with constraint dependence, 90 population sizes were initially taken, while for the survival of the next generation, the elite count in the reproduction option was put on default. Moreover, for genetic variation and also to achieve new individual formation, constraint dependent mutation and crossover functions were applied (Table S3). A Lagrangian solver was adopted for better accuracy as the nonlinear constraint algorithm, whereas Design Expert software was adopted to achieve the optimal condition by keeping the independent variables in their maximum range. Table 4 depicts the optimized process results for RSM-GA, RSM-DF and ANN-GA. ANN-GA and RSM-DF showed higher values in comparison with RSM-GA. All of the three methods exhibited similar values; however, the ANN-GA conditions were further used to validate the model and make the comparison. The final extraction was also carried out by modifying the conditions as follows: ethanol concentration (50%), extraction time (3 min), extraction temperature (140 °C), and power (600 W) for further confirmation, and the results are presented in Table S2.

### 3.4. Cell Viability, ROS Scavenging, and Antioxidant Enzyme Activity

**Figure 2:**
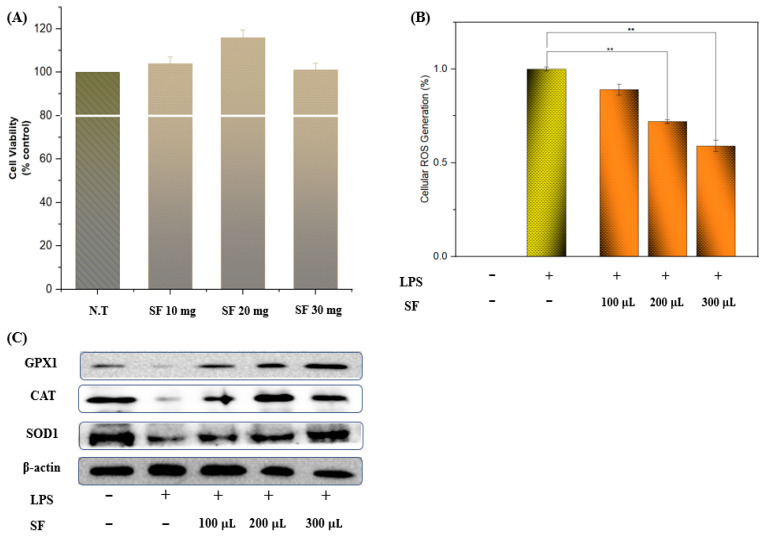
Cellular viability and antioxidant activity of optimized *Sargassum fusiforme* (SF) extracts. Cell viability of optimized SF extracts (**A**), ROS scavenging activity of SF extracts (**B**), glutathione peroxidase 1 (GPx-1), catalase (CAT), and superoxide dismutase 1 (SOD-1) protein expression (**C**) in RAW cells were measured as described in materials and methods. Results are expressed as the mean ± SD of three separate experiments. LPS: 1 μg/mL. ** *p* < 0.01 as compared with LPS alone.

SF extracts obtained after optimizing the MAE conditions did not exhibit any RAW 264.7 cellular toxicity up to 300 µg/mL (Figure 2A). Figure 2B shows that ROS production was enhanced by LPS induction; however, SF extracts scavenged ROS in a dose-dependent manner (approximately two-fold). Similarly, SF extracts also increased antioxidant enzyme activity, that is, catalase (CAT), glutathione peroxidase-1 (GPx-1), and superoxidase-1 (SOD-1) levels in a concentration-dependent pattern (Figure 2C). Wang et al. (2022) stated that fermented fucoidan isolated from SF attenuates ROS levels and improves SOD levels in zebrafish induced by H_2_O_2_ [35], which is consistent with our results.

### 3.5. Metabolite Profiling of SF Extracts Using High-Resolution LC-MS/MS

Metabolite profiling of the optimized SF extract (Supplementary Table S2) was conducted in the negative mode of HRLC-ESI-MS/MS. As shown in Table 5, 79 secondary metabolites were tentatively verified, and 12 of them were reported for the first time in SF, which were as follows.

#### 3.5.1. Phenolic Acids

During collisions, phenolic acids exhibit certain breakage patterns and loss-specific molecules, such as methyl (15 Da) and carboxyl (44 Da) [36]. Based on the fragmentation pattern, compounds **1**–**2** were previously identified as gallic acid and vanillic acid, respectively [21]. Compound **3**, reported in *Dasya* sp., yielded two daughter ions at m/z 179.03 (isopropanol group) and m/z 123.04 by cleaving the α, β carbon–carbon bond, and was identified as isopropyl 3-(3,4-dihydroxyphenyl)-2-hydroxypropanoate [37]. Compound **4** (3,4-dihydroxyphenylglycol) yielded a precursor ion at m/z 169.0506, which was further fragmented into two major daughter ions at m/z 151.03 (losing H_2_O) and 123.00 (losing CO). Similarly, compound **5** was previously reported in *Codium* and *Grateloupia* sp., and produced a parent ion at m/z 191.0352 and was fragmented further by losing methyl (15 Da) ([M-H-CH_3_]) and carboxyl (44 Da) ([M-H-CH_3_-COO]) at m/z 175.03 and 147.04, respectively [38]. Compound **6**, with a monoisotopic mass [M-H]^−^ at m/z 258.9912, forms fragmentation ions at m/z 215.00 by losing [M-H-CH_3_-COO], followed by further cleavage into a caffeic acid ion (135.04 Da) by losing SO_3_ (80 Da). Fragmentation of phenolic acid glycoside started at the glycosidic link and yielded a sugar moiety (−162 Da), which tentatively confirmed the presence of compound **7** (cinnamoyl glucose) in SF by having a characteristic ion at m/z 303.08 [38]. Compounds **3–7** have been reported for the first time in SF extracts.

#### 3.5.2. Flavonoids

Compound **8**, previously known as 3′-O-methylcatechin, produced an ion peak at m/z 303.0868 with characteristic peaks at m/z 271.07 and m/z 163.05, corresponding to a loss of 30 Da (CH_3_O) and 109 Da (C_6_H_5_O_2_), respectively, identified for the first time in this species. Compound **9**, previously known as 4’,7-dihydroxyisoflavone, had a precursor ion peak at m/z 253.0500 and fragment peaks at m/z 225.05 and m/z 197.06, owing to the loss of two CO molecules [M-H-2CO], followed by the removal of water molecules from the B ring at m/z 143.03. Similarly, compound **10** (naringenin) was previously found in *Ulva intestinalis* and tentatively identified by Choi et al. [21]. Naringenin exhibits various biological properties, including anti-inflammatory and anticancer activities [39]. Compounds **9** and **10** were also reported for the first time in SF-optimized extracts.

#### 3.5.3. Tannins and Terpenoids

Compounds **11**–**16** were tentatively identified as tannins and compounds **17**–**21** as terpenoids, as shown in Table 5. Tannins have a complex structure and, in seaweeds, this complexity pertains specifically to phlorotannins, including phlorethols, fuhalos, fucols, and eckols. In the SF extract, compounds **11**–**14** were previously identified as phloroglucinol, fucophlorethol-A, bifuhalol, and diphlorethohydroxycarmalol, respectively [19,40]. Diphlorethohydroxycarmalol exhibits protective effects against oxidative stress in retinal pigment epithelial cells and was tentatively reported for the first time in SF extracts [41]. Compound **15**, previously identified in *Fucus* sp., yielded a monoisotopic mass [M-H]^−^ at m/z 246.9914 with corresponding minor peaks at m/z 202 and 121, indicating that the compound was dibenzodioxin-1,3,6,8-tetraol, which was reported for the first time in SF extracts [42]. Similarly, compound **16** (fucophlorethol), previously reported in *Laminaria digitate,* was identified for the first time in SF extracts based on the fragment pattern reported by [43]. Compounds **17**–**20** were previously identified as glycyrrhizin, β-glycyrrhetinic acid, isololiolide, and lupenone, respectively, in SF [44,45].

#### 3.5.4. Carboxylic Acid, Fatty Acids, Sugars, and Amino Acids

Compounds **21**–**35** were previously reported in a variety of seaweeds and were identified as carboxylic acids based on their fragmentation patterns. Compounds **36**–**66** were fatty acids, compounds **67**–**69** were amino acids, and compounds **70**–**74** were sugars [30,46,47,48,49,50,51], as shown in Table 5. Additionally, compounds **75** and **76** (phenols), and compound **77** (alcohol) were identified. Compound **78** was previously identified in *Ascophyllum nodosum* as dihydroxyphenylalanine, based on its fragmentation pattern. Dihydroxyphenylalanine is a large neutral amino acid with a catecholamine precursor that has a neuroprotective effect, especially against Parkinson’s disease [52]. Compound **79** (5-hydroxybenzofuran-2(3H)-one) yielded a monoisotopic mass at m/z 149.0238, which represented an aromatic alcohol group. To the best of our knowledge, compounds **78** and **79** have been tentatively reported for the first time for SF.

## 4. Conclusions

In this study, the effects of MAE parameters on the antioxidant potential of *Sargassum fusiforme* were investigated using two sophisticated techniques: RSM and ANN-GA. A comparative overview of both statistical models based on SEP, RMSE, R^2^, and AAD revealed that the ANN-GA models were superior to the RSM model. The optimized MAE conditions (*X*_1_: 47.67%, *X*_2_: 2.96 min, *X*_3_: 139.54 °C, and *X*_4_: 600.00 W) exhibited maximum response for four dependent variables: Y_1_:28.01% DPPH inhibition; Y_2_:36.07% ABTS inhibition; Y_3_:43.65 mg GAE/g TPC; Y_4_:17.67 mg CAE/g TFC content. The optimized extracts showed no toxicity to RAW 264.7 cells up-to 300 µg/mL and had increased CAT, GPx-1, and SOD-1 levels in a dose-dependent manner. After optimizing the MAE conditions, optimized extracts were analyzed by HR-LC-MS and identified 79 secondary metabolites; among them, 12 new bioactive compounds (five phenolic compounds, two flavonoids, three tannins, and two other compounds) were also tentatively reported for the first time in optimized SF extracts. The current investigation may not only provide an alternative statistical technique, but it could also support the preferred extraction technology for identifying important bioactive components that can be used in broad commercial applications as promising ingredients for the development of functional foods and nutraceuticals and in the cosmetic industry, shedding light on the bright side of human health. However, several other aspects that might affect the extractability of bioactive components, including kinetic behavior, type of solvent, pH, ultrasound frequency, and quantitative measurement of bioactive components, should be investigated, as this study provides the basis for further future trials.

## Figures and Tables

**Table 1:** Central composite design (CCD) for independent variables and corresponding target responses (experimental).

Run No	Parameter				DPPH (% Inhibition)			ABTS (% Inhibition)			TPC (mg GAE/g)			TFC (mg CAE/g)		
	X_1_ (%)	X_2_ (min)	X_3_ (°C)	X_4_ (W)	Experimental Value	RSM Predicted	ANN Predicted	Experimental Value	RSM Predicted	ANN Predicted	Experimental Value	RSM Predicted	ANN Predicted	Experimental Value	RSM Predicted	ANN Predicted
1	70	4	140	600	18.94 ± 2.98	20.34	19.30	37.14 ± 0.54	38.87	37.08	33.88 ± 0.62	33.77	33.45	17.67 ± 0.16	18.15	17.68
2	50	3	130	150	26.13 ± 3.12	25.84	25.15	28.30 ± 2.13	28.51	28.20	25.40 ± 0.62	25.78	25.51	10.61 ± 0.44	9.67	9.96
3	30	2	110	300	20.95 ± 1.12	19.89	21.13	36.25 ± 1.87	34.58	36.20	29.55 ± 0.17	29.80	29.51	12.89 ± 0.65	13.36	12.90
4	50	3	130	450	25.75 ± 2.98	26.79	25.62	40.83 ± 1.35	41.80	41.61	36.02 ± 2.15	35.58	35.44	14.49 ± 0.16	14.03	14.47
5	10	3	130	450	17.42 ± 1.29	19.86	20.49	29.00 ± 2.34	31.46	28.96	22.29 ± 0.17	23.68	23.76	10.61 ± 0.33	9.61	10.60
6	90	3	130	450	7.31 ± 1.61	6.39	6.43	25.36 ± 1.77	23.30	26.08	21.50 ± 1.24	20.81	21.49	9.23 ± 0.42	8.45	9.33
7	50	3	130	450	26.13 ± 2.35	26.79	25.62	41.83 ± 1.35	41.80	41.61	35.05 ± 0.79	35.58	35.44	13.49 ± 0.95	14.03	14.47
8	50	3	90	450	14.76 ± 1.64	16.51	15.45	30.37 ± 1.51	31.39	30.26	21.44 ± 1.37	21.26	21.60	7.50 ± 0.75	7.50	7.51
9	30	4	110	300	23.39 ± 2.41	21.79	23.45	34.01 ± 2.07	34.03	34.68	30.55 ± 0.75	28.52	29.96	11.61 ± 0.88	11.42	11.60
10	30	4	140	300	20.76 ± 2.32	21.00	20.18	29.40 ± 3.70	28.78	29.39	20.48 ± 1.72	21.83	20.88	7.45 ± 0.65	8.40	7.44
11	50	3	130	750	27.63 ± 2.61	29.44	28.90	42.61 ± 0.63	42.80	42.49	35.99 ± 0.52	36.31	36.85	18.95 ± 0.44	18.11	18.90
12	70	2	140	600	19.58 ± 2.20	19.53	19.64	34.20 ± 2.40	34.47	32.69	26.99 ± 2.25	28.54	26.76	16.85 ± 0.36	17.82	17.13
13	30	2	140	600	29.21 ± 2.60	29.48	28.40	42.29 ± 2.38	40.85	42.16	30.94 ± 0.17	29.27	30.85	16.65 ± 0.38	17.60	17.45
14	30	4	140	600	32.59 ± 1.12	29.20	31.87	39.39 ± 3.39	39.50	39.31	31.44 ± 1.64	30.23	31.59	13.19 ± 1.10	13.90	13.18
15	70	2	110	300	13.97 ± 2.03	15.08	13.22	25.61 ± 2.02	25.61	25.66	20.74 ± 2.03	21.51	20.74	6.44 ± 1.01	6.47	6.46
16	50	3	130	450	27.25 ± 1.75	26.79	25.62	41.83 ± 1.35	41.80	41.61	36.08 ± 0.34	35.58	35.44	13.49 ± 0.58	14.03	14.47
17	50	5	130	450	27.71 ± 2.62	28.68	29.97	38.89 ± 1.59	37.40	38.84	28.55 ± 1.20	27.74	28.67	12.45 ± 0.98	11.80	12.43
18	70	4	140	300	16.77 ± 2.11	17.89	15.43	28.19 ± 1.90	29.89	28.21	23.41 ± 0.12	23.44	23.56	9.24 ± 0.45	9.96	9.32
19	30	2	140	300	30.79 ± 3.26	31.07	30.40	32.37 ± 1.35	31.97	32.25	25.23 ± 1.30	23.89	27.72	11.82 ± 0.65	12.54	11.78
20	70	2	110	600	11.96 ± 2.20	11.85	11.66	27.48 ± 0.97	27.40	24.64	22.63 ± 0.60	21.06	22.61	6.94 ± 0.74	7.00	8.54
21	70	4	110	300	18.51 ± 0.79	18.08	19.66	30.64 ± 1.78	30.82	30.56	23.41 ± 0.89	24.49	25.33	8.43 ± 0.68	8.55	8.43
22	30	2	110	600	24.84 ± 1.20	22.39	27.18	38.94 ± 1.05	38.11	38.90	27.56 ± 1.64	27.43	27.64	11.21 ± 0.77	11.21	11.21
23	50	1	130	450	29.42 ± 2.33	29.96	27.75	32.54 ± 1.60	34.43	32.56	22.55 ± 0.62	24.06	22.56	15.28 ± 0.36	14.15	15.28
24	70	4	110	600	26.77 ± 1.15	24.64	26.70	34.49 ± 2.10	34.44	32.63	26.55 ± 0.37	27.06	25.76	9.42 ± 0.12	9.54	9.44
25	30	4	110	600	32.74 ± 1.55	34.09	32.34	39.87 ± 1.16	39.39	40.81	27.88 ± 1.26	29.18	28.04	9.52 ± 0.45	9.71	9.72
26	50	3	150	450	27.71 ± 2.30	26.24	27.96	39.33 ± 0.64	37.81	39.19	27.67 ± 0.66	28.95	27.73	15.35 ± 0.64	15.97	15.38
27	70	2	140	300	27.44 ± 1.44	28.85	27.89	27.36 ± 2.30	27.33	27.32	22.80 ± 0.76	21.24	22.72	9.23 ± 0.39	10.08	9.31

**Table 2:** ANVOA analysis for well-fitted RSM model.

**Source**	DF	DPPH	ABTS	TPC	TFC
**Model**	14	1068.95 ***	804.11 ***	635.00 ***	302.80 ***
**Intercept (*β*_0_)**	1	26.34	41.84	35.80	13.26
**Linear terms**					
**X_1_ (*β*_1_)**	1	0.6782	0.9626 *	−0.9867 **	−0.4032 **
**X_2_ (*β*_2_)**	1	1.72 **	0.4558	0.1990	1.67 ***
**X_3_ (*β*_3_)**	1	−3.42 ***	−2.40 ***	−1.19 **	−0.6597 **
**X_4_ (*β*_4_)**	1	1.24 *	3.13 ***	1.99 ***	1.51 ***
**Quadratic terms**					
**X_1_^2^ (*β*_11_)**	1	0.6328	−1.47 ***	−2.42 ***	0.1636
**X_2_^2^ (*β*_22_)**	1	−1.07 *	−1.72 ***	−2.58 ***	−0.4717 **
**X_3_^2^ (*β*_33_)**	1	−3.42 ***	−3.60 ***	−3.33 ***	−1.15 ***
**X_4_^2^ (*β*_44_)**	1	0.2118	−1.54 ***	−1.13 ***	0.0652
**Interaction terms**					
**X_1_X_2_ (*β*_1_*β*_2_)**	1	−2.99 ***	−0.6606	−0.1952	−0.5511 **
**X_1_X_3_ (*β*_1_*β*_3_)**	1	0.2740	1.44 *	1.06 **	1.01 ***
**X_1_X_4_ (*β*_1_*β*_4_)**	1	2.45 ***	0.4581	0.7562	0.1127
**X_2_X_3_ (*β*_2_*β*_3_)**	1	0.1471	1.0 8*	1.41 **	1.11 ***
**X_2_X_4_ (*β*_2_*β*_4_)**	1	−1.02	1.34 **	1.94 ***	1.80 ***
**X_3_X_4_ (*β*_3_*β*_4_)**	1	−1.43 **	−0.4356	0.4797	0.6715 **
**Lack of fit (probability)**	10	0.1138	0.0981	0.1025	0.3556
**F-value**		8.18	9.58	9.15	2.18
**R^2^**		0.9543	0.9608	0.9533	0.9745
**Adj. R^2^**		0.9009	0.9151	0.8989	0.9447
**Adeq. precision**		17.9849	15.8235	12.9272	19.0817
**C.V. (%)**		8.90	4.81	5.90	6.86

The level of significance is expressed as *** *p* < 0.001, ** *p* < 0.01, and * *p* < 0.05.

**Table 3:** Comparison of the prediction abilities of the RSM and ANN models.

Parameters	DPPH		ABTS		TPC		TFC	
	RSM	ANN	RSM	ANN	RSM	ANN	RSM	ANN
RMSE	1.38	1.15	1.10	0.78	1.15	0.56	0.66	0.46
R2	95.43	96.84	96.08	98.03	95.33	97.72	96.27	98.19
AAD	5.18	4.17	2.40	1.26	3.52	1.68	4.66	2.12
SEP	0.22	0.18	0.12	0.08	0.15	0.10	0.21	0.14

**Table 4:** Process optimization using three different methods.

Variables		RSM-GA	ANN-GA	RSM-DF (0.92)
Independent variables	Concentration	47.75	47.67	47.36
	Time	2.76	2.96	2.94
	Temperature	140.79	139.54	139.71
	Power (W)	599.99	600.00	599.99
Dependent variables	DPPH	26.31	28.01	27.94
	ABTS	35.99	36.07	36.00
	TPC	42.90	43.65	43.58
	TFC	17.84	17.67	17.63

**Table 5:** Identified compounds of the optimized extract of *Sargassum fusiforme* by ESI-MS/MS.

Group	No.	Compound Name	EF	CM (m/z)^−/+^	OM (m/z)^−/+^	MS/MS (Negative Mode)
Phenolic acids and derivatives	**1.**	Gallic acid	C_7_H_6_O_5_	169.0134	169.0137	125.02
	**2.**	Vanillic acid	C_8_H_8_O_4_	167.0343	167.0344	108.02, 152.011
	**3.**	Isopropyl 3-(3,4-dihydroxyphenyl)-2-hydroxypropanoate ^#^	C_12_H_16_O_5_	239.0929	239.0925	173.03, 123.04
	**4.**	3,4-Dihydroxyphenylglycol ^#^	C_8_H_10_O_4_	169.0500	169.0506	151.03, 123.00
	**5.**	Scopoletin ^#^	C_10_H_8_O_4_	191.0349	191.0350	175.03, 147.04
	**6.**	Caffeic acid 4-Sulfate ^#^	C_9_H_8_O_7_S	258.9899	258.9912	215.00, 179.03, 135.04
	**7.**	Cinnamoyl Glucose ^#^	C_15_H_18_O_7_	309.0974	309.0974	147.04, 131.04, 103.05
Flavonoids	**8.**	3’-O-Methylcatechin	C_16_H_16_O_6_	303.0862	303.0868	271. 07, 163.05
	**9.**	4’,7-Dihydroxyisoflavone ^#^	C_15_H_10_O_4_	253.0493	253.0500	225.05,197.06, 143.03
	**10.**	Naringenin ^#^	C_15_H_12_O_5_	271.0581	271.0606	229.05, 177.01, 151.00, 119.06
Tannins	**11.**	Phloroglucinol	C_6_H_6_O_3_	125.0237	125.0238	97.04
	**12.**	Fucophlorethol-A	C_18_H_14_O_9_	373.0557	373.0559	247.05, 233.02, 229.07, 125.03
	**13.**	Bifuhalol	C_12_H_10_O_7_	264.0345	264.0348	247.03, 141.07, 111.03, 123.01, 125.03,
	**14.**	Diphlorethohydroxycarmalol ^#^	C_24_H_15_O_13_	511.0510	511.0506	385.00
	**15.**	Dibenzodioxin-1,3,6,8-tetraol ^#^	C_12_H_7_O_6_	246.9919	246.9914	203.05, 121.01
	**16.**	Fucophlorethol ^#^	C_36_H_26_O_14_	680.1154	680.1179	610.05,601.03, 495.07, 469.06, 229.03
Terpenes	**17.**	Glycyrrhizin	C_42_H_62_O_16_	821.3951	821.3959	777.40, 627.35, 469.33, 451.32
	**18.**	β-Glycyrrhetinic acid	C_30_H_46_O_4_	469.3330	469.3317	451.32, 423.32, 409.31
	**19.**	Isololiolide	C_11_H_16_O_3_	195.1021	195.1021	161.09, 179.10, 133.10, 105.07
	**20.**	Lupenone	C_30_H_48_O	423.3620	423.3626	408.33,381.33,365.27,257.24
Carboxylic acids	**21.**	Fumaric acid	C_4_H_4_O_4_	115.0050	115.0037	71.01
	**22.**	Threonic acid	C_4_H_8_O_5_	135.0290	135.0299	117.01, 91.04, 72.99
	**23.**	Cinnamic acid	C_9_H_8_O_2_	147.0442	147.0452	129.03, 103.05
	**24.**	Gentisic acid	C_7_H_6_O_4_	153.0187	153.1287	152.74, 108.07, 81.05
	**25.**	Behenic acid	C_22_H_44_O_2_	339.3268	339.3263	321.31, 295.33,211.24
	**26.**	Kainic acid	C_10_H_15_NO_4_	212.0922	212.0922	168.2, 194.1, 150.2
	**27.**	Mannuronic acid	C_6_H_10_O_7_	193.0353	193.0348	175.02, 103.00, 72.99
	**28.**	Diethyl phthalate	C_12_H_14_O_4_	221.0818	221.0813	193.08, 177.09, 149.09, 121.02
	**29.**	Vanillylmandelic acid	C_9_H_10_O_5_	197.0449	197.0450	153.05, 137.02,123.04, 107.01
	**30.**	Phthalic acid	C_8_H_6_O_4_	165.0188	165.0187	121.1 26, 119.15, 58.91
	**31.**	3-Oxooctanoic acid	C_8_H_14_O_3_	157.0863	157.0876	139.07, 113.09, 97.06
	**32.**	D-Glucaric acid derivate	C_12_H_14_O_10_	317.0544	317.0509	209.08
	**33.**	Fukic acid	C_11_H_12_O_8_	271.0459	271.0453	227.05, 197.04, 179.03
	**34.**	Mono-(3-carboxypropyl) phthalate	C_12_H_12_O_6_	251.0556	251.0555	233.04, 207.06, 165.01
	**35.**	Azelaic acid	C_9_H_16_O_4_	187.0969	188.0976	187.31, 124.91, 169.20, 111.20
Fatty acids	**36.**	Caprylic acid	C_8_H_15_O_2_	143.1070	143.1072	125.09, 99.11, 59.01
	**37.**	Stearic acid	C_18_H_36_O_2_	283.2641	283.2637	265.25, 239.27, 237.25
	**38.**	cis-Vaccenic acid	C_18_H_34_O_2_	281.2486	281.2487	263.23, 223.17, 163.14, 71.01
	**39.**	α-Linoleic acid	C_18_H_32_O_2_	279.2331	279.2330	261.22, 235.24, 233.22
	**40.**	Oleic acid	C_18_H_34_O_2_	281.2487	281.2486	263.25, 181.21, 127.25
	**41.**	Palmitic acid	C_16_H_32_O_2_	255.233	255.233	237.23, 211.24, 197.22
	**42.**	Myristic acid	C_14_H_28_O_2_	227.2015	227.2017	209.19, 183.21,179.18
	**43.**	Arachidic acid	C_20_H_40_O_2_	311.2958	311.295	297.04, 275.84, 200.85
	**44.**	Eicosapentaenoic acid	C_20_H_30_O_2_	301.2176	301.2173	299.20, 283.20, 229.15, 131.08, 71.01
	**45.**	Arachidonic acid	C_20_H_32_O_2_	303.2333	303.233	285.2218, 259.24, 257.22
	**46.**	5,8,11-Eicosatrienoic acid	C_20_H_34_O_2_	305.2491	305.2486	287.23, 261.25, 207.21
	**47.**	11,14-Eicosadienoic acid	C_20_H_36_O_2_	307.2643	307.2643	289.25, 249.18, 233.19, 153.09, 71.01
	**48.**	Octadecendioic acid	C_18_H_32_O_4_	311.223	311.2239	299.25, 269.24, 251.23, 223.24
	**49.**	Methyl arachidonate	C_21_H_34_O_2_	317.2487	317.2486	315.23, 301.21, 243.17, 191.18, 121.10, 73.02
	**50.**	13-keto-9Z,11E-Octadecadienoic acid	C_18_H_30_O_3_	293.2125	293.2122	275.20, 195.13, 113.09
	**51.**	10-Oxooctadecanoic acid	C_18_H_34_O_3_	297.2436	297.2435	279.23, 209.15, 141.12, 127.11
	**52.**	10,16-Dihydroxy-palmitic acid	C_16_H_32_O_4_	287.2227	287.2222	269.21, 257.21, 239.20, 185.11
	**53.**	Vernolic acid	C_18_H_32_O_3_	295.2278	295.2273	277.21, 251.23, 195.13, 127.11
	**54.**	Octadeca-2,4-dienedioic acid	C_18_H_30_O_4_	309.2072	309.2071	291.19, 265.21, 247.20
	**55.**	Palmitaldehyde	C_16_H_32_O	239.092	239.0925	237.22, 207.21, 153.12, 127.14
	**56.**	Myristic aldehyde	C_14_H_28_O	211.2064	211.2067	209.19, 167.14, 127.14, 99.11, 71.08
	**57.**	Heptanal	C_7_H_14_O	113.0962	113.0972	95.08, 85.10
	**58.**	9-Octadecenal	C_18_H_34_O	265.2544	265.2537	249.22, 247.24, 235.24
	**59.**	2,4-Decadienal	C_10_H_16_O	151.1119	151.1128	133.10, 123.11, 119.08, 93.07
	**60.**	Nonanal	C_9_H_18_O	141.128	141.1285	123.11, 113.13, 95.08
	**61.**	Ethyl oleate	C_20_H_38_O_2_	309.2798	309.2799	291.26, 281.24, 263.23, 237.25, 45.03
	**62.**	1-Docosanol	C_22_H_46_O	325.3475	325.3476	309.31, 307.33, 295.33, 267.30
	**63.**	(9R,10S,12Z)-9,10-Dihydroxy-8-oxo-12-octadecenoic acid	C_18_H_32_O_5_	327.2178	327.2177	309.20, 283.22, 187.09, 157.08
	**64.**	5,8,12-Trihydroxy-9-octadecenoic acid	C_18_H_34_O_5_	329.2338	329.2333	311.22, 285.24, 267.23, 243.12, 195.17, 145.08
	**65.**	13-Docosenamide	C_22_H_43_NO	336.3274	336.3272	319.30, 293.3214, 58.02
	**66.**	Lauric acid	C_12_H_24_O_2_	199.1697	199.1698	181.1062; 155.0336
Amino Acids	**67.**	L-Proline	C_5_H_9_NO_2_	114.0560	114.0555	70.06
	**68.**	Glutamic acid	C_5_H_9_NO_4_	146.0451	146.0453	70.06
	**69.**	D-Histidine	C_6_H_9_N_3_O_2_	154.0628	154.0616	82.3, 71.9
Sugars	**70.**	D-Galactose	C_6_H_12_O_6_	179.0568	179.0555	161.04, 143.03, 113.02, 101.02,
	**71.**	Mannitol	C_6_H_14_O_6_	181.0712	181.0718	165.01, 147.03, 129.05, 111.00
	**72.**	Gluconic acid	C_6_H_12_O_7_	195.0517	195.0504	177.05, 159.02, 129.05, 98.90
	**73.**	Xylitol	C_5_H_12_O_5_	151.0618	151.0612	119, 131, 89.1
	**74.**	Maltitol	C_12_H_24_O_11_	343.1248	343.124	283.10, 265.09, 179.05, 161.04, 143.03
Other compounds	**75.**	4-Octylphenol	C_14_H_22_O	205.1593	205.1592	135.08, 119.05, 107.05, 93.03
	**76.**	2,4-Dibromophenol	C_6_H_4_Br_2_O	248.8649	248.8550	248.85, 168.92
	**77.**	Dihydroconiferyl alcohol	C_10_H_14_O_3_	181.0864	181.0864	163.07, 135.04
	**78.**	Dihydroxyphenylalanine ^#^	C9H10O7NS	276.0185	276.0184	259, 231, 215, 196, 179, 150, 135
	**79.**	5-Hydroxybenzofuran-2(3H)-one ^#^	C_8_H_6_O_3_	149.0237	149.0238	121.02, 67.01, 65.00

EF: elemental formula; OM: observed mass; CM: calculated mass; (-): Negative mode; ^#^ First time identification in SF.

## Data Availability

Data are contained within the article and supplementary materials.

## Supplementary Materials

The following supporting information can be downloaded at: https://www.mdpi.com/article/10.3390/antiox11112246/s1. Table S1: Independent process variables with experimental ranges and levels for MAE of SF, Table S2: Comparison between optimized model and experimental values, Table S3. Setting parameters of genetic algorithm used in the optimization of process for SF, Figure S1: The three-dimensional (3D) response surface plots of MAE-SF extraction condition displaying the influence of independent parameters (Ethanol Concentration, Time, Temperature, and power) on dependent variables (DPPH radical-scavenging activity, ABTS, TPC, and TFC) as a function of significant interaction factors for RSM, Figure S2: Regression of experimental and predicted values in ANN model of MAE for SF using the training, testing and validation datasets to optimized the extraction conditions.

## Abbreviations

AAD, absolute average deviation; ABTS, 2,2′-Azino-Bis-3-Ethylbenzothiazoline-6-Sulfonic acid; ANN, artificial neural network; β, regression coefficient; BFGS, Broyden–Fletcher–Goldfarb–Shanno algorithm; trainlm, Levenberg—Marquardt backpropagation algorithm; CAE, catechin equivalent; CCD, central composite design; CNS, central nervous system; CV, coefficient of variation; DI, desirability index; DPPH, 1,1-Diphenyl-2-picrylhydrazyl; GA, genetic algorithm; GAE, gallic acid equivalent; HR-LC-MS, high-resolution liquid chromatography-mass spectrometry; MLP, multilayer perceptron network; MSE, mean square error; R^2^, adjusted correlation factor; RMSE, root mean squared error; RSSFM, response surface methodology; SEP, standard error of prediction; SF, *Sargassum fusiforme* (Harvey) Setchell; TFC, total flavonoid content; TPC, total phenolic content; MAE, Microwave-assisted extraction.
