# Supplementary material for: Metabolite Profiling of Microwave-Assisted Sargassum fusiforme Extracts with Improved Antioxidant Activity Using Hybrid Response Surface Methodology and Artificial Neural Networking-Genetic Algorithm
Source: Antioxidants (Basel). 2022 Nov 14;11(11):2246. doi: 10.3390/antiox11112246 (PMC9687032; doi:10.3390/antiox11112246)
Supplement: Supplementary file 1 [file antioxidants-11-02246-s001.zip › antioxidants-1997227-supplementary.pdf]

**Supplementary materials:**

**Table S1**: Independent process variables with experimental ranges and levels for MAE of SF

| Variables | Factors | Level | | | | |
|---|---|---|---|---|---|---|
| | **$X_i$** | **$\alpha$-1** | **-1** | **0** | **1** | **$\alpha$+1** |
| **Ethanol concentration (%)** | $X_1$ | 0 | 30 | 50 | 70 | 90 |
| **Extraction time (min)** | $X_2$ | 1 | 2 | 3 | 4 | 5 |
| **Extraction temperature (°C)** | $X_3$ | 90 | 110 | 130 | 140 | 150 |
| **Equipment power** | $X_4$ | 150 | 300 | 450 | 600 | 750 |

**Table S2**: Comparison between optimized model and experimental values

| **Dependent variables** | **Experimental value** | **ANN-GA** |
|---|---|---|
| DPPH (% inhibition) | 29.23±0.41 | 28.017 |
| ABTS (% inhibition) | 35.49±0.19 | 36.075 |
| TPC (mgGAE/g) | 43.03±0.24 | 43.658 |
| TFC (mgCAE/g) | 17.30±0.08 | 17.679 |

**Figure S1.** The three-dimensional (3D) response surface plots of MAE-SF extraction condition displaying the influence of independent parameters (Ethanol Concentration, Time, Temperature, and Intensity) on dependent varaiables (DPPH radical-scavenging activity, ABTS, TPC, and TFC) as a function of significant interaction factors for RSM.

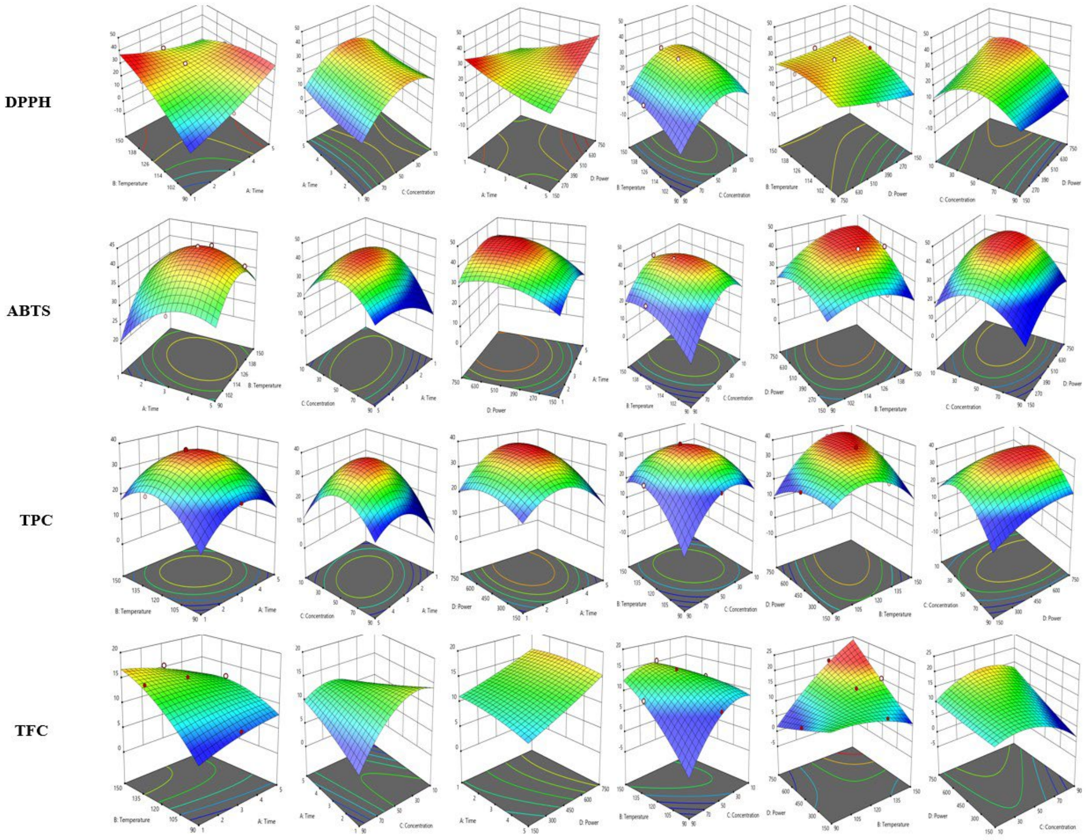


**Figure S2:** Regression of experimental and predicted values in ANN model of MAE for SF using the training, testing and validation datasets to optimized the extraction conditions.

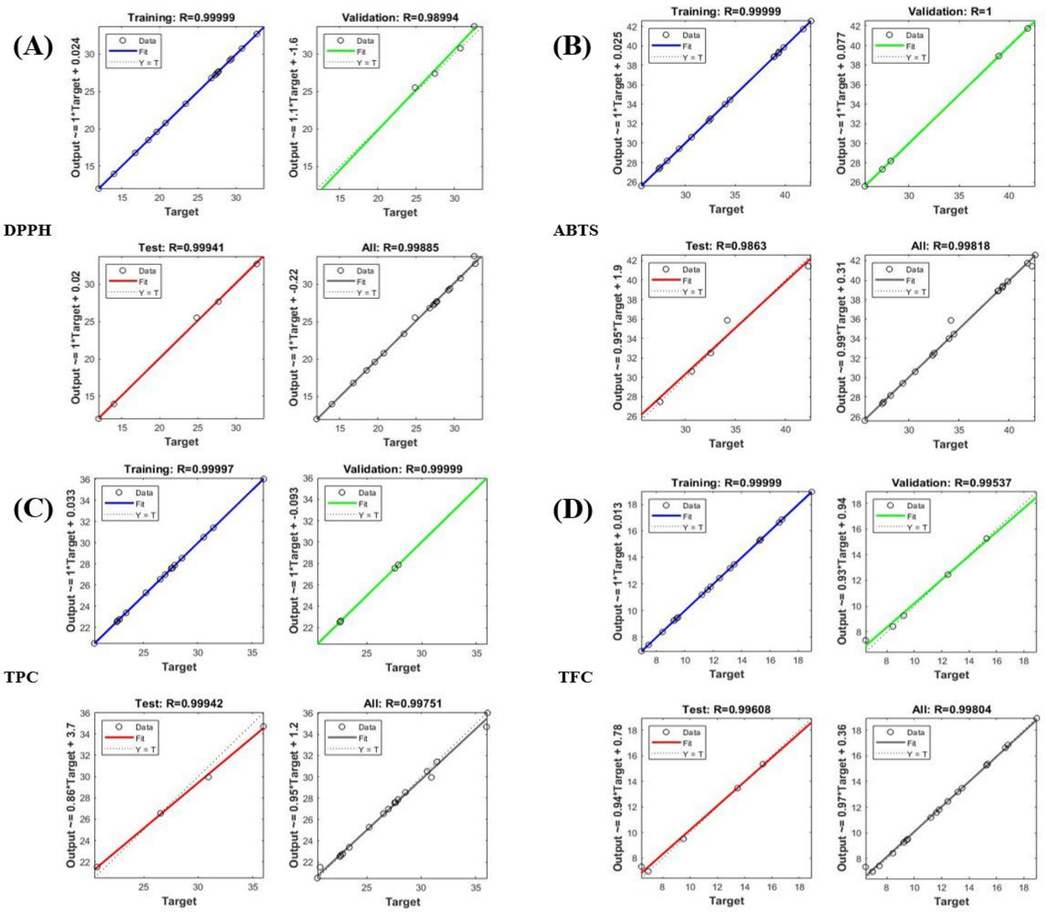


**Table S3**. Setting parameters of genetic algorithm used in the optimization of process for SF

| Setting parameters | Values |
|---|---|
| **Population size** | 90 |
| **Scaling function** | Rank |
| **Selection function** | Stochastic uniform |
| **Elite count** | default |
| **Crossover function** | Constraint dependent |
| **Mutation function** | Constraint dependent |
| **Plot function** | Best fitness |
| **Nonlinear constraint algorithm** | Augmented Lagrangian |
